# Two for the GOES: Exploring Gambling Outcome Expectancies Scores Across Mixed and Offline-Only Gamblers in Relation to Problem Gambling Risk Status

**DOI:** 10.1007/s10899-023-10234-x

**Published:** 2023-06-27

**Authors:** Andrea C. Richardson, Mal Flack, Kim M. Caudwell

**Affiliations:** 1 School of Human Services, Faculty of Health, Charles Darwin University, Ellengowan Drive, Casuarina, NT 0810 Australia; 2 Researchers in Behavioural Addictions, Alcohol and Drugs, Charles Darwin University, Ellengowan Drive, Casuarina, NT 0810 Australia

**Keywords:** Gambling, Online gambling, Motivation, Outcome expectancies, Gambling-related harm

## Abstract

As online gambling becomes more prevalent, understanding the motives of online gamblers has become a key focus for research and practice. The aim of this study was to understand differences in gambling-related outcome expectancies between mixed (both online and offline) gamblers and offline-only gamblers, by incorporating gambling harm risk categories from the problem gambling severity index (PGSI). This study comprised a secondary data analysis of the 2015 Northern Territory Gambling Prevalence and Wellbeing Survey. A sample of 1207 individuals in the Northern Territory who had reported gambling at least once in the previous 12 months were used in the analyses. General linear and structural equation modelling were used to ascertain differences in gambling outcome expectancies, in relation to gambling modality (i.e., mixed, offline-only) and PGSI scores. Mixed gamblers tended to score higher on all outcome expectancies than their offline-only counterparts. Outcome expectancy scores were higher in individuals in higher-risk PGSI categories. The *escape* outcome expectancy was dependent on both modality and risk category. Invariance testing of a low and problem gambling risk subsample revealed differential relationships for both the escape and excitement outcome expectancies for mixed and offline-only gamblers. The results provide an important contribution to the existing literature regarding motivation and outcome expectancies in relation to gambling modality and problem gambling severity. The findings highlight the importance of considering both gambling outcome expectancies and modality when considering problem gambling.

## Introduction

Gambling has long been a popular pastime, but has become increasingly available in various online formats from the early to mid-1990s (Wood & Williams, 2011). Although a smaller proportion of gamblers gamble online (Dowling et al., 2015; Gainsbury et al., 2015), the prevalence is increasing (Abbott et al., 2018). Approximately half of online gamblers will also engage in other methods of gambling (Goldstein et al., 2016), meaning that gamblers can be categorised as exclusively online, exclusively offline (i.e., land-based), or engaging in both online and offline (i.e., mixed) forms of gambling (Gainsbury et al., 2015). Recent research by Hing et al. (2022) estimates nearly 70% of Australian gamblers are land-based only, with 8.1% online-only, and 22.6% mixed mode (i.e., gambling online and offline). Much is known about the impact of different gambling activities on the individual in relation to the experience of gambling-related harm. For instance, gamblers who use Electronic Gaming Machines (EGMs), play casino table and card games (e.g., poker), and engage in sports betting, are more likely to experience gambling-related harm compared to lotteries players and horse race bettors (Binde, 2011; Binde et al., 2017). Concerningly, increased accessibility of gambling can also increase risk of gambling problems, with longitudinal studies finding that the most consistent predictors of gambling problems are gambling participation measures, such as frequency of gambling, gambling expenditure, and gambling duration (Abbott et al., 2018).

Many of the aforementioned factors that contribute to risk of gambling problems are present in online gambling. Of particular concern is that online gambling is highly accessible, usually solitary, and is not associated with conventional avenues for intervention by gambling venue staff (e.g., ability for players to use credit, lack of age verification and ability to play multiple games simultaneously; Abbott et al., 2018; Dowling et al., 2015). Those who participate in online gambling tend to have a higher risk of experiencing gambling problems (Goldstein et al., 2016; Stevens et al., 2019), however, research has found mixed results when comparing gamblers by type (Gainsbury et al., 2015). Recently, Tomei et al. (2022) found that the proportion of gamblers in the at-risk/problem gambling category was larger for mixed mode gamblers than those in the non-problem category (i.e., fewer offline gamblers comprised the at-risk/problem gambling proportion). Similarly, mixed-method gamblers are overrepresented in problem gambling compared to online-only and land-based gamblers (Hing et al., 2022). An important area of inquiry is to therefore understand the motivations or expectancies of gamblers in relation to their gambling behaviour by modality, as well as in relation to problem gambling status.

A range of motivational and belief-based facets appear to underlie gambling behaviour, including the obvious potential for financial gain, but also various interpersonal and affective motives or expectancies (Binde, 2013; Flack & Morris, 2015; Neighbors et al., 2002). Several motive-based measures have been used to assess reasons for gambling, including the gambling motivation scale (GMS; Chantal et al., 1994) and gambling motives questionnaire (GMQ; Stewart & Zack, 2008). An alternative way to assess reasons for gambling are outcome expectancies (Flack & Morris, 2015), which do not conflate reasons for gambling with gambling frequency. In general, differences in reasons for gambling have been found to differentiate subgroups of gamblers. For instance, Canale et al. (2015) found mixed gamblers scored higher on enhancement, recreation, and money motives than their offline counterparts. Goldstein et al. (2016) found that online gamblers scored significantly higher on coping motives than did offline-only gamblers, but that no significant differences were found between the two groups on enhancement and social motives. Further, respondents were significantly more likely to initiate online gambling to make money, pass time, and demonstrate skill; whereas, offline gambling was initiated for reasons such as excitement, feeling lucky, being sociable, or for performative reasons (e.g., beating someone. Dowling et al. (2015) found that gambling for positive feelings was predictive of both past year gambling and frequent (i.e., monthly) online gambling. Reasons for gambling have also been investigated in relation to problem gambling status. For instance, *money*, *social*, and *ego* outcome expectancies have been shown to predict gambling frequency, whereas *ego*, *excitement*, and *escape*, predict problem gambling severity (Flack & Morris, 2015). The *escape* and *excitement* subscales of the Gambling Outcome Expectancies Scale (GOES; Flack & Morris, 2015) were also predictive of problem gambling at 12-month follow-up (Flack & Morris, 2016).

Some research has looked at how different reasons for gambling may be associated with problem gambling status. For instance, Lloyd et al. (2010) reported that online gamblers experiencing gambling problems were more likely to rate highly on money as well as enjoyment and mood-regulation motivations than non-problem gamblers. Mulkeen et al. (2017) found that online gamblers who were experiencing gambling problems scored higher on money and excitement motivations, whereas those not experiencing gambling problems scored higher on escape and relaxation motives. More recently, Greer et al. (2022) and Lelonek-Kuleta and Bartczuk (2021) found gambling to escape or improve mood and financial motivation were positively associated with problem gambling severity for e-sport bettors.

### The Present Study

Taken together, these findings suggest financial and emotion-oriented motivational factors are positively associated with the experience of problem gambling. However, most of the research on gambling motives and outcome expectancies in relation to gambling behaviour and outcomes has focused on gambling modality (i.e., offline vs. online) or has evaluated the associations between motives and risk of problem gambling. It is likely that across different gambling modes, individuals will hold different outcome expectancies, and this may vary as a function of problem gambling status. For example, a low-risk online-only gambler may consider gambling as a good way to relax (e.g., at home), whereas a low-risk offline gambler may consider gambling to be an enjoyable experience (e.g., while at a casino), but problem gamblers may gamble offline or in mixed modality to escape. As such, investigating the outcome expectancies of gamblers who engage in different modalities, at varying levels of problem gambling severity, may yield important information for gambling interventions (Kok et al., 2016). For instance, gamblers who exhibit strong escape motives may be identified and approached for gambling-related counselling or self-exclusion from gambling venues (Yi et al., 2015). Similarly, evidence for expectancy challenge approaches in the context of alcohol consumption has demonstrated efficacy in relation to modifying expectancies and reducing alcohol consumption and alcohol-related harm (Gesualdo & Pinquart, 2021). The present study therefore investigates gambling outcome expectancies by risk level and gambling modality (i.e., offline vs. mixed).

### Methods

#### Participants

This study involved secondary data analysis of the 2015 Northern Territory Gambling Prevalence and Wellbeing Survey.^1^ The sample included 4945 adults aged 18 years or older who resided in the Northern Territory. A report that describes the methodological and sampling parameters in more detail has been published elsewhere (Stevens et al., 2017).

### Measures

#### Severity of Gambling Problems

The Problem Gambling Severity Index (Ferris & Wynne, 2001) was used to assess severity of gambling problems. The PGSI has nine items (e.g., “thinking about the last 12 months, have you bet more than you could really afford to lose?”), with responses scored on a 4-point Likert-type scale, from 0 (“never”) to 3 (“almost always”). The PGSI scoring paradigm categorises gamblers in to four groups: non-risk, low risk, moderate risk, and problem gamblers. Due to the low number of participants in the moderate-risk and problem gambler categories, these were combined to an “at-risk” category", consistent with the findings of previous investigations PGSI risk categories (e.g., Currie et al., 2013) and as used in previous research (e.g., Flack & Stevens, 2019). The PGSI demonstrated adequate reliability in the survey (Cronbach’s α = .81).

#### Gambling Outcome Expectancies

The Gambling Outcome Expectancy Scale (GOES; Flack & Morris, 2015) assesses beliefs about gambling that comprise five dimensions (i.e., *excitement*, *escape*, *ego*, *social*, and *money*). Each of the 18-items are rated on a 6-point Likert-type scale from 1 (“*strongly disagree”*) to 6 (“*strongly agree*”), although for the 2015 Gambling Prevalence and Wellbeing Survey, the scale was modified to 5-point response option for ease of verbal administration. Item scores were averaged within subscales. The GOES subscales have demonstrated good internal reliability (Cronbach’s α range = .85 to .94) and temporal stability (Flack & Morris, 2016). Confirmatory Factor Analysis confirmed the five-factor structure of the GOES in the dataset (Flack & Stevens, 2019).

### Procedure

Participant responses were recorded by Roy Morgan Research and Associates using a Computer Aided Telephone Interviewing (CATI) method, following a random digit dialling approach. Respondents were selected based on the ‘last birthday’ method, until the strategy was altered to allow for more male respondents. For participants contacted via mobile phone, the owner of the phone was considered the respondent. Further information on the methodology used in the survey can be found in the published report (Stevens et al., 2017). Participants provided sociodemographic information, and those who indicated they had gambled within the past 12 months (*N* = 1207; 24.4%) completed the GOES.

### Statistical Analyses

In relation to gambling modality, participants were categorised as “mixed” and “offline-only” gamblers, depending on their responses to questions related to online gambling over the previous 12-month period. Participants who selected “online” for any question were considered “mixed” (*n* = 138; 11.4%) as they may have also gambled offline, whereas those who did not select “online” for any question were categorised as “offline-only” (*n* = 1069; 88.6%). This is consistent with Hing et al. (2022) who found the vast majority of people who gamble online also gamble offline.

In relation to the PGSI, 66.3% of participants were categorised non-risk gamblers, 23.9% were low-risk gamblers and the remaining 9.8% were grouped together in the at-risk category. The ages of the participants ranged between 18 and 89 years (*M*_age_ = 48.9 years; *SD*_age_ = 14.1 years). The sample consisted of 44.6% male, 7.5% Indigenous, 95.9% who spoke English as the main language at home, 76.9% who were employed at the time, and 80.4% who completed year 12 education or higher (see Table 1 for comparisons by gambling modality).

**Table 1:** Proportions and mean differences across gambler type group

Category/variable	Offline only	Mixed	*p* value^b^
Non-risk gamblers	70.0%	37.7%	< .001
Low-risk gamblers	22.3%	37.0%	
At-risk gamblers	7.8%	25.4%	
Male	45.0%	71.0%	< .001
Indigenous	7.1%	10.9%	.120
Speak English at home	95.7%	97.8%	.300
Employed	75.8%	86.2%	< .001
Education^a^	80.0%	84.1%	.260
*M*_age_ (*SD*_Age_)	50.1 (13.9)	43.5 (13.1)	< .001

^a^Completion of Year 12 or above; ^b^χ^2^ or *t*-test

### General Linear Model Analyses

SPSS version 28 was used for analyses. To test for the interaction between PGSI risk level (.e., no risk, low risk, at risk) and gambling modality (i.e., mixed, offline-only), a series of univariate General Linear Models (GLMs) were conducted, with the GOES outcome expectancies as outcome variables. To test for the interaction between PGSI risk level (i.e., no risk, low risk, at risk) and gambling modality (i.e., mixed, offline-only), a series of univariate General Linear Models (GLMs) were conducted, with the GOES outcome expectancies as outcome variables. Given the cell sizes and resulting F-max value (2.08), we adopted a conservative alpha level (.01) with which to assess the significance of interactions (Tabachnik & Fidell, 2012).

### Invariance Testing

A related focus of the present study was to evaluate whether the pattern of relationships observed between GOES subscales and PGSI score were consistent across both gambling modalities (i.e., offline and mixed). Participants who were included in the at-risk and problem gambler categories were combined to test the invariance of a path model using structural equation modeling (SEM), whereby each motive dimension from the GOES was used to predict PGSI score. The unconstrained SEM formed as a baseline, after which the constraint of metric invariance for gambling modality was imposed (i.e., paths were constrained to equality). Significant incremental change in model (χ^2^) would therefore indicate a different pattern of relationships between gambling outcome expectancies for online and mixed gamblers. SPSS AMOS was used for analyses, using the Gaskin and Lim (2018) multigroup analysis plugin.

### Ethics

Access to data was provided by the Northern Territory Department of Health, with ethical approval granted by the Charles Darwin University Human Research Ethics Committee (HREC Approval Number: H20025).

## Results

### General Linear Model Analyses

**Fig. 1:**
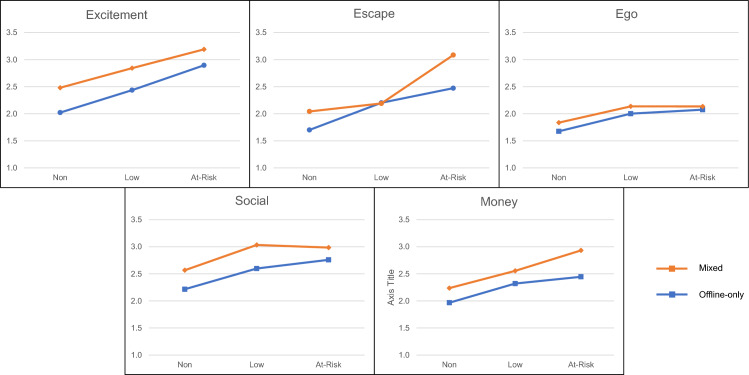
Means plots for each of the GOES subscales, by PGSI category, between mixed and offline-only gamblers. A significant interaction effect was observed for the Escape subscale only (*p* = .002)

Across the five GOES subscales, a significant interaction was observed for the *escape* subscale only *F*(2, 1201) = 6.37, *p* = .002, *η*^2^_p_ = .01. Simple effects revealed that at the non-risk level of problem gambling severity, the difference between mixed (*M* = 2.04, *SE* = .10) and offline-only gamblers (*M* = 1.70, *SE* = .03) was statistically significant (*p* < .001); as for those in the at-risk PGSI category (*mixed*: *M* = 3.09, *SE* = .12; *offline-only*: *M* = 2.47, *SE* = .08; *p* < .001). All other interaction effects for the *excitement*, *ego*, *social*, and *money* subscales were non-significant (*F*s < .67; *p*s > .513). Means plots for GLMs are included in Fig. 1.

Main effects for gambling modality were statistically significant for four of the five GOES dimensions: *excitement F*(1,1201) = 23.52, *p* < .001, *η*^2^_p_ = .02; *escape*, *F*(1,1201) = 20.60, *p* < .001, *η*^2^_p_ = .02; *social*, *F*(1,1201) = 15.29, *p* < .001, *η*^2^_p_ = .01; and, *money*, *F*(1,1202) = 14.24, *p* < .001, *η*^2^_p_ = .01. Follow up simple comparisons with Bonferroni corrections indicated mixed gamblers scored higher on these dimensions than their offline-only counterparts. No significant differences were observed for *ego; F*(1,1201) = 3.69, *p* = .055, *η*^2^_p_ < .01.

Main effects for gambling problems were statistically significant for all five GOES dimensions: *excitement*, *F*(2, 1201) = 31.64, *p* < .001, *η*^2^_p_ = .05; *escape*, *F*(1,1201) = 52.98, *p* < .001, *η*^2^_p_ = .08; *ego*, *F*(2,1201) = 14.93, *p* < .001, *η*^2^_p_ = .02; *social*, *F*(2, 1201) = 14.24, *p* < .001, *η*^2^_p_ = .02; and, *money*, *F*(2,1201) = 15.12, *p* < .001, *η*^2^_p_ = .02. Follow-up simple comparisons with Bonferroni corrections indicated participants in higher risk categories tended to score higher across these subscales, though there was no significant differences between low risk and at-risk participant scores for *ego* (*p* = 1.000), *social* (*p* = 1.000), and *money* (*p* = .085).

### Invariance Testing

**Fig. 2:**
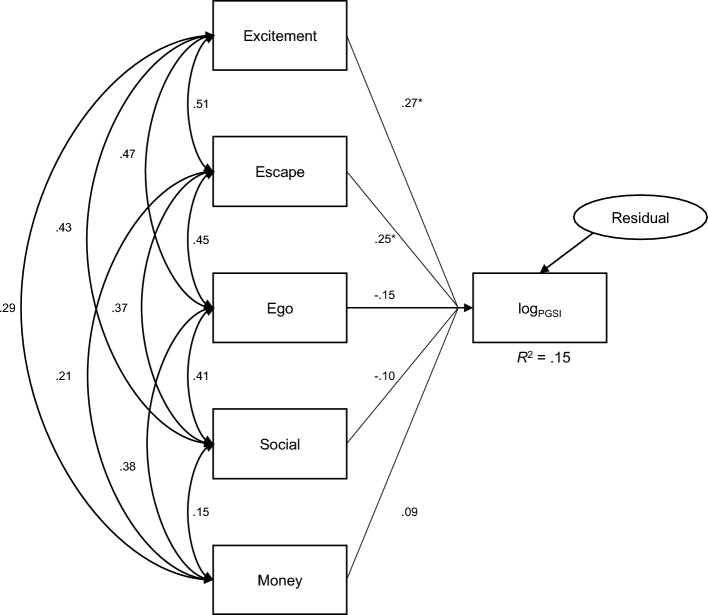
Path model showing standardised estimates from GOES subscales to log-transformed PGSI scores. *p < .05

A subsample comprising participants in low and at-risk categories was tested using structural equation modelling in AMOS. Given the skew of the raw PGSI data, PGSI scores were log transformed. An unconstrained path model that comprised paths from each GOES subscale to log-transformed PGSI scores was fitted to the data (see Fig. 2).

For the subsample, the excitement and escape paths were statistically significant. Subsequent imposition of equality constraints to test metric invariance led to a significant reduction in model fit; ∆χ^2^ (5) = 13.43, *p* = .020. Observation of the structural paths indicated that regression coefficients were dissimilar across the mixed and offline-only groups. Investigation of model path results revealed freeing both the *excitement* and *escape* paths led to a non-significant change in model fit over the unconstrained model, supporting partial invariance. Specifically, for the offline-only group, the *excitement* path was a significant, positive predictor of PGSI scores, whereas *escape* was non-significant; in the mixed modality group, the *escape* path was a significant positive predictor of PGSI scores, whereas *excitement* was non-significant. A summary of the model change fit statistics and unstandardised parameter estimates are reported in Table 2.

**Table 2:** Results of invariance testing and predictors by gambling modality category

Invariance test	Model comparison	∆χ^2^	∆*df*	∆*p*	Freed parameters	
1. Unconstrained	–	–	–	–	All parameters	
2. Metric invariance	2 vs. 1	13.43	5	.020		
3. Partial invariance	3 vs. 1	1.95	3	.583	Excite → PGSI	
					Escape → PGSI	

Predictors	Gambling modality category					
	Total (*n* = 407)		Offline (*n* = 321)		Mixed (*n* = 86)	
	*β*	SE	*β*	SE	*β*	SE
Excitement	.07**	.02	.08**	.02	.04	.04
Escape	.07**	.02	.03	.02	.15**	.03
Ego	− .05	.02	− .04	.02	− .08	.04
Social	− .03	.01	− .02	.02	− .03	.03
Money	.02	.01	− .01	.01	− .05	.03
*R*^2^	.15**		.10**		.35**	

*p < .05; **p < .01

## Discussion

The present study used existing data from the 2015 Northern Territory Gambling Prevalence and Wellbeing Survey to investigate the associations between gambling expectancies, problem gambling severity, and gambling modality. While several studies have looked at the differences in motivation between gamblers in relation to gambling modality, or by problem gambling severity status, the present study aimed to contribute further to this literature by looking at (1) how gambling outcome expectancies may differ across both gambling mode and gambling risk severity, and (2) whether or not expectancies differ in how they predict problem gambling severity based on gambling modality.

Analyses indicated a significant interaction between the *escape* subscale only. Specifically, mixed modality gamblers scored higher on this dimension than offline-only gamblers at the non-risk and at-risk levels of problem gambling severity. It is interesting to note that there was no significant difference in means for escape at the low risk level of problem gambling severity. One tentative interpretation of this finding is that non-risk mixed gamblers who score higher in escape outcome expectancies may be more likely to progress to at-risk gambling if these expectancies continue to correspond with their gambling behaviour over time (Flack & Morris, 2016). Similarly, a higher escape score in the at-risk mixed mode gambler group may be reflective of at-risk gamblers feeling compelled to engage across various gambling modes as they experience increased gambling-related harm (Sleczka & Romild, 2021; Tseng et al., 2023). Notably, the mean difference between mixed and offline-only gamblers for at-risk gamblers was nearly twice that for non-risk level gamblers (i.e., .34 and .61 respectively). Generally speaking, these findings suggest that mixed gamblers are more motivated to gamble for reasons related to escape, and that this is dependent on level of problem gambling severity.

Investigation of the main effects indicated a trend whereby higher problem gambling severity scores were associated with higher mean item scores on each of the GOES subscales; and that mixed gambling modality was associated with higher mean item scores on the GOES subscales (with the exception of the *ego* subscale). These findings indicate three things: that mixed-modality gamblers are more motivated to gamble for reasons of escape; that higher problem gambling severity is generally associated with higher motivations to gamble for different reasons; and that gambling motivation is generally higher in those who engage in mixed types of gambling. These findings are broadly consistent with other motive-based research. For instance, research by Dowling et al. (2015) and Goldstein et al. (2016) has found no differences between groups on social and money motive dimensions. Goldstein et al. (2016) found no difference in relation to enhancement (akin to excitement), but that online gamblers scored higher on coping motivations, which are similar to the escape expectancies from the GOES. Although these findings are consistent with the interpretation of the interaction effect in the present study, reconciling the present findings with existing research is difficult. For example, the Reasons for Gambling Questionnaire (Francis et al., 2015) was developed in a United Kingdom gambling context, but the factor structure was not confirmed when tested in an Australian sample. This is likely due to the differences in gambling regulations, advertisement, accessibility, and cultural perceptions of gambling on gambling experiences, expectancies, and behaviours (Gainsbury & Wood, 2011; Wood & Williams, 2011). Similarly, the conceptualisation of gambling type is also important. For instance, Dowling et al. (2015) did not include EGMs as forms of online gambling, though individuals can access EGMs online, with some accessing prohibited online ‘offshore’ gambling sites (Gainsbury et al., 2018).

Although some studies have shown social reasons to be more prominent in relation to offline gambling than online gambling (Goldstein et al., 2016; McCormack & Griffiths, 2012), findings revealed that mixed gamblers scored higher on the social outcome expectancy than offline-only gamblers. This inconsistency may be due to online gambling having more of a social aspect than previously considered. Indeed, a recent systematic review has shown that online communities provide opportunities for gamblers to connect with like-minded others and therefore serve a social connection role in parallel with online gambling (Sirola et al., 2021). Though in the past, online gambling websites included basic tools for social exchange (e.g., chat functions; McCormack & Griffiths, 2012), betting companies have made good use of social media platforms to promote sports gambling in particular (Thomas et al., 2015), posting information about special offers, gambling wins by customers, and links to sports teams (Gainsbury et al., 2016). Indeed, a recent systematic review of sports betting has indicated a range of influential and persuasive marketing and promotion initiatives seek to normalise sports betting (Etuk et al., 2022). Ultimately, that mixed gamblers scored higher on social outcome expectancies challenges notions of gamblers and warrants future research of how this manifests in predominantly online gamblers.

The invariance testing method used indicated that the associations between GOES subscales and PGSI score were partially invariant with regard to gambling modality. Overall, the model showed significant prediction by the escape and excitement expectancies, however when the modality constraint was imposed, model fit improved after allowing the *excitement* and *escape* paths to vary (meaning that these paths had different associations with problem gambling severity depending on the sample). Namely, for mixed gamblers, *escape* is predictive of problem gambling severity but *excitement* is not; whereas for offline gamblers, the opposite pattern emerges. To speculate, individuals may opt to gamble across multiple modes because of circumstances in their life that lead them to psychologically escape, or cope, with these circumstances. Indeed, research has indicated that online gambling is more closely aligned with endorsing coping motives (Goldstein et al., 2016; Greer et al., 2022; Lelonek-Kuleta & Bartczuk, 2021). These findings suggest gambling modality is important to consider when investigating the relationship between gambling motives and problem gambling behaviour, and that individuals gambling across different modalities (i.e., mixed gamblers) may need tailored interventions to reduce gamblng-related harm (Dowling et al., 2015; Goldstein et al., 2016; Griffiths et al., 2009).

Overall, these findings are particularly important for two reasons: (1) the continued increase in the popularity of online gambling, and online availability of traditionally offline gambling products; and (2) challenges in relation to facilitating access to gambling interventions for gamblers who engage more in the online gambling space. For instance, research has illustrated that online gamblers exhibit lower rates of uptake across a variety of support initiatives (Hing et al., 2015). Similarly, a recent review has indicated that online gambling communities may serve to reinforce excessive gambling behaviours as gamblers engage in these communities to celebrate wins and share tips (Savolainen et al., 2022). Of concern, too, are findings from recent reseach on esports betting—an exclusively online form of gambling—that suggests players exhibit similar affect-regulation motives when engaging in cash betting, skin betting, and skin gambling activities, and that these are linked to problem gambling (Greer et al., 2022). Given the proliferation of ‘loot boxes’ in online games targeted at children and young adults, and links between purchase behaviour, gambling problems, and positive gambling-related attitudes more generally (Rockloff et al., 2021), more research is needed to tease out the complex relationship between gambling type, modality, and motives, in their relationship to gambling-related harm.

### Strengths and Limitations

The main strength of the present study is its focus on gambling outcome expectancies in relation to both gambling modality and problem gambling severity. This approach enabled the modeling of interactions between these variables in relation to gambling motive, leading to the observation that the *escape* subscale was particularly relevant in discerning offline-only and mixed gamblers, at two of the three levels of problem gambling severity. Similarly, inclusion of these two variables allowed for a finer-grained analysis of the predictive relationships between the GOES susbcales and PGSI score by gambling modality in low and at-risk gamblers. To the authors’ knowledge, this is the first study to compare these associations at differing levels of gambling using an invariance testing approach; without this approach, the differential influences of escape and excitement motives would not have been observed. Future research should continue to probe the nature of the relationship between motives and gambling-related outcomes, to ascertain the boundary conditions under which these predictive relationships hold. Such research would be important to inform ‘just-in-time’ adaptive interventions that have recently been developed to tailor intervention approaches (e.g., “exploring expectancies”) to gamblers in an attempt to prevent excessive or problematic gambling episodes (Dowling et al., 2022).

An additional strength of the present study lies in the use of prevalence study data, collected via a robust CATI methodology. This enabled the analysis of a sample that can be considered broadly representative of gamblers in the Northern Territory (Stevens et al., 2017). Although the findings are based on a sample of NT gamblers, they can are broadly consistent with the findings of National prevalence studies. For instance, the rate of problem gambling based on PGSI scores in the prevalence study was .7%, within the .5–1% observed in a study by the Australian Productivity Commission (2010). Additionally, the demographic profiles of mixed and offline only gamblers were consistent with that observed in previous studies; for example, mixed gamblers were younger, more likely to be male, and employed (Griffiths et al., 2009; McCormack & Griffiths, 2012; Wood & Williams, 2011).

Of course, there are some limitations of the present study that should be noted when considering the findings. Firstly, the inclusion criteria for gamblers was that the participant had engaged in gambling activity at least once in the past year. Although this is a common means of screening participants through the gambling literature (Gainsbury et al., 2013; Griffiths et al., 2009), it is challenging in that there are a variety of gambling types that could be engaged in with greater frequency, that would preclude more insidious but less frequent forms of gambling (Dowling et al., 2015). Secondly, the intent of the prevalence survey to capture a representative sample of gamblers within the Northern Territory meant that fewer online gamblers were captured, which meant samples sizes differed (i.e., *n* = 138 online gamblers vs. *n* = 1069 offline-only gamblers). Targeting participants via online gambling-related websites has led to samples with a higher proportion of online gamblers (e.g., Gainsbury et al., 2013; McCormack & Griffiths, 2012), however introduces a challenge in relation to selection bias and generalisability. The use of a mixed group in this study was intended to better balance the group sizes, however likely represents three types of gamblers: (a) mainly offline gamblers who supplement their gambling with occasional online gambling, (b) mainly online gamblers who occasionally gamble offline, and (c) those who gamble exclusively online. Although it is assumed that mixed gamblers can be substituted for online gamblers when compared with offline only gamblers, future gambling research should attempt to untangle mixed mode gamblers, which could be acheived by using more targeted sampling methods.

## Conclusion

This study provides an important contribution to our understanding of gambling motivations, modality, and problem gambling. Reconciling these areas of inquiry has revealed findings that have important implications for public health and policy attempts to reduce gambling-related harm, and efforts to challenge expectancies. Notably, a recent report by the Australia Media Communications Authority (ACMA; 2022) has observed the extensive use of social media by gambling companies and affiliates to attract and retain gamblers, with online strategies including promoting ‘tipsters’ and contributing and creating gambling communities. Such strategies can potentially contribute to the development of expectancies that are linked with problem gambling (Sirola et al., 2018). Regardless of where problem gambling is likely to occur, reducing gambling-related harm will not be easy if the reasons for gambling, and gambling modality, are only considered in isolation.

## Data Availability

The data supporting the findings in the current study are available from the corresponding author upon reasonable request.

## Footnotes

1 *Note*. The most recent Northern Territory Gambling Prevalence Survey (2018) did not include the GOES, and as such, the 2015 version was used.

## References

[CR1] Abbott, M. W., Binde, P., Clark, L., Hodgins, D. C., Johnson, M. R., Manitowabi, D., Quilty, L. C., Spangberg, J., Volberg, R. A., Walker, D. M., & Williams, R. J. (2018). *Conceptual framework of harmful gambling* (3rd ed.). Gambling Research Exchange Ontario. 10.1556/2006.2020.00024 PMC8939413 32554839

[CR2] Australian Communications and Media Authority. (2022). The role of affiliate services in promoting illegal online gambling in Australia: Desktop research. https://www.acma.gov.au/sites/default/files/2022-02/

[CR3] Australian Government Productivity Commission. (2010). *Gambling*. Australian Government Productivity Commission. https://www.pc.gov.au/inquiries/completed/gambling-2010/report

[CR4] Binde, P. (2011). *What are the most harmful forms of gambling? Analyzing problem gambling prevalence surveys*. CEFOS Working Papers 12. CEFOS.

[CR5] Binde, P. (2013). Why people gamble: A model with five motivational dimensions. *International Gambling Studies,**13*(1), 81–97. 10.1080/14459795.2012.712150 10.1080/14459795.2012.712150

[CR6] Binde, P., Romild, U., & Volberg, R. A. (2017). Forms of gambling, gambling involvement and problem gambling: Evidence from a Swedish population survey. *International Gambling Studies,**17*(3), 490–507. 10.1080/14459795.2017.1360928 10.1080/14459795.2017.1360928

[CR7] Canale, N., Vieno, A., Griffiths, M. D., Rubaltelli, E., & Santinello, M. (2015). How do impulsivity traits influence problem gambling through gambling motives? The role of perceived gambling risk/benefits. *Psychology of Addictive Behaviors,**29*(3), 813–823. 10.1037/adb0000060 25730629 10.1037/adb0000060

[CR8] Chantal, Y., Vallerand, R. J., & Vallères, E. F. (1994). Assessing motivation to gamble: On the development and validation of the Gambling Motivation Scale. *Loisir Et Société / Society and Leisure,**17*(1), 189–212. 10.1080/07053436.1994.10715471 10.1080/07053436.1994.10715471

[CR9] Currie, S. R., Hodgins, D. C., & Casey, D. M. (2013). Validity of the problem gambling severity index interpretive categories. *Journal of Gambling Studies,**29*, 311–327. 10.1007/s10899-012-9300-6 22426971 10.1007/s10899-012-9300-6

[CR10] Dowling, N. A., Lorains, F. K., & Jackson, A. C. (2015). Are the profiles of past-year internet gamblers generalizable to regular internet gamblers? *Computers in Human Behavior,**43*, 118–128. 10.1016/j.chb.2014.10.019 10.1016/j.chb.2014.10.019

[CR100] Dowling, N. A., Merkouris, S. S., Youssef, G. J., Lubman, D. I., Bagot, K. L., Hawker, C. O., ... & Rodda, S. N. (2022). A gambling just-in-time adaptive intervention (gamblingless: in-the-moment): protocol for a microrandomized trial. *JMIR Research Protocols*, *11*(8), e38958. 10.2196/38958 10.1016/j.chb.2014.10.019 PMC9449828 35998018

[CR11] Etuk, R., Xu, T., Abarbanel, B., Potenza, M. N., & Kraus, S. W. (2022). Sports betting around the world: A systematic review. *Journal of Behavioral Addictions*. 10.1556/2006.2022.00064 36067022 10.1556/2006.2022.00064 PMC9872539

[CR12] Ferris, J. A., & Wynne, H. J. (2001). *The Canadian problem gambling index*. http://jogoremoto.pt/docs/extra/TECb6h.pdf

[CR13] Flack, M., & Morris, M. (2015). Problem gambling: One for the money…? *Journal of Gambling Studies,**31*(4), 1561–1578. 10.1007/s10899-014-9484-z 24986780 10.1007/s10899-014-9484-z

[CR14] Flack, M., & Morris, M. (2016). The temporal stability and predictive ability of the Gambling Outcome Expectancies Scale (GOES): A prospective study. *Journal of Gambling Studies,**32*(3), 923–933. 10.1007/s10899-015-9581-7 26518686 10.1007/s10899-015-9581-7

[CR15] Flack, M., & Stevens, M. (2019). Gambling motivation: Comparisons across gender and preferred activity. *International Gambling Studies,**19*(1), 69–84. 10.1080/14459795.2018.1505936 10.1080/14459795.2018.1505936

[CR16] Francis, K. L., Dowling, N. A., Jackson, A. C., Christensen, D. R., & Wardle, H. (2015). Gambling motives: Application of the reasons for Gambling Questionnaire in an Australian Population Survey. *Journal of Gambling Studies,**31*(3), 807–823. 10.1007/s10899-014-9458-1 24705633 10.1007/s10899-014-9458-1

[CR17] Gainsbury, S., & Wood, R. (2011). Internet gambling policy in critical comparative perspective: The effectiveness of existing regulatory frameworks. *International Gambling Studies,**11*(3), 309–323. 10.1080/14459795.2011.619553 10.1080/14459795.2011.619553

[CR18] Gainsbury, S. M., Delfabbro, P., King, D. L., & Hing, N. (2016). An exploratory study of gambling operators’ use of social media and the latent messages conveyed. *Journal of Gambling Studies,**32*(1), 125–141. 10.1007/s10899-015-9525-2 25644444 10.1007/s10899-015-9525-2

[CR19] Gainsbury, S. M., Russell, A., Hing, N., Wood, R., & Blaszczynski, A. (2013). The impact of internet gambling on gambling problems: A comparison of moderate-risk and problem Internet and non-Internet gamblers. *Psychology of Addictive Behaviors,**27*, 1092–1101. 10.1037/a0031475 23438251 10.1037/a0031475

[CR20] Gainsbury, S. M., Russell, A., Hing, N., Wood, R., Lubman, D., & Blaszczynski, A. (2015). How the internet is changing gambling: Findings from an Australian Prevalence Survey. *Journal of Gambling Studies,**31*(1), 1–15. 10.1007/s10899-013-9404-7 23934369 10.1007/s10899-013-9404-7 PMC4611023

[CR21] Gainsbury, S. M., Russell, A. M., Hing, N., & Blaszczynski, A. (2018). Consumer engagement with and perceptions of offshore online gambling sites. *New Media & Society,**20*(8), 2990–3010. 10.1177/1461444817738783 10.1177/1461444817738783

[CR22] Gaskin, J., & Lim, J. (2018). *Multigroup analysis*. http://statwiki.gaskination.com/

[CR23] Gesualdo, C., & Pinquart, M. (2021). Expectancy challenge interventions to reduce alcohol consumption among high school and college students: A meta-analysis. *Psychology of Addictive Behaviors,**35*(7), 817. 10.1037/adb0000732 33856837 10.1037/adb0000732

[CR24] Goldstein, A. L., Vilhena-Churchill, N., Stewart, S. H., Hoaken, P. N., & Flett, G. L. (2016). Mood, motives, and money: An examination of factors that differentiate online and non-online young adult gamblers. *Journal of Behavioral Addictions,**5*(1), 68–76. 10.1556/2006.5.2016.003 28092184 10.1556/2006.5.2016.003 PMC5322999

[CR25] Greer, N., Hing, N., Rockloff, M., Browne, M., & King, D. L. (2022). Motivations for esports betting and skin gambling and their association with gambling frequency, problems, and harm. *Journal of Gambling Studies*. 10.1007/s10899-022-10137-3 35802281 10.1007/s10899-022-10137-3 PMC9981487

[CR26] Griffiths, M., Wardle, H., Orford, J., Sproston, K., & Erens, B. (2009). Sociodemographic correlates of internet gambling: Findings from the 2007 British Gambling Prevalence Survey. *CyberPsychology & Behavior,**12*(2), 199–202. 10.1089/cpb.2008.0196 19072080 10.1089/cpb.2008.0196

[CR27] Hing, N., Russell, A. M. T., Black, A., Rockloff, M., Browne, M., Rawat, V., Greer, N., Stevens, M., Dowling, N. A., Merkouris, S., King, D. L., Salonen, A. H., Breen, H., & Woo, L. (2022). Gambling prevalence and gambling problems amongst land-based-only, online-only and mixed-mode gamblers in Australia: A national study. *Computers in Human Behavior,**132*, 107269. 10.1016/j.chb.2022.107269 10.1016/j.chb.2022.107269

[CR28] Hing, N., Russell, A. M. T., Gainsbury, S. M., & Blaszczynski, A. (2015). Characteristics and help-seeking behaviors of Internet gamblers based on most problematic mode of gambling. *Journal of Medical Internet Research,**17*(1), e3781. 10.2196/jmir.3781 10.2196/jmir.3781 PMC4296092 25567672

[CR29] Kok, G., Gottlieb, N. H., Peters, G.-J.Y., Mullen, P. D., Parcel, G. S., Ruiter, R. A. C., Fernández, M. E., Markham, C., & Bartholomew, L. K. (2016). A taxonomy of behaviour change methods: An intervention mapping approach. *Health Psychology Review,**10*(3), 297–312. 10.1080/17437199.2015.1077155 26262912 10.1080/17437199.2015.1077155 PMC4975080

[CR30] Lelonek-Kuleta, B., & Bartczuk, R. P. (2021). Online gambling activity, pay-to-win payments, motivation to gamble and coping strategies as predictors of gambling disorder among e-sports bettors. *Journal of Gambling Studies,**37*(4), 1079–1098. 10.1007/s10899-021-10015-4 33689100 10.1007/s10899-021-10015-4 PMC8572820

[CR31] Lloyd, J., Doll, H., Hawton, K., Dutton, W. H., Geddes, J. R., Goodwin, G. M., & Rogers, R. D. (2010). How psychological symptoms relate to different motivations for gambling: An online study of internet gamblers. *Biological Psychiatry,**68*(8), 733–740. 10.1016/j.biopsych.2010.03.038 20655512 10.1016/j.biopsych.2010.03.038

[CR32] McCormack, A., & Griffiths, M. D. (2012). Motivating and inhibiting factors in online gambling behaviour: A grounded theory study. *International Journal of Mental Health and Addiction,**10*(1), 39–53. 10.1007/s11469-010-9300-7 10.1007/s11469-010-9300-7

[CR33] Mulkeen, J., Abdou, H. A., & Parke, J. (2017). A three stage analysis of motivational and behavioural factors in UK internet gambling. *Personality and Individual Differences,**107*, 114–125. 10.1016/j.paid.2016.11.007 10.1016/j.paid.2016.11.007

[CR34] Neighbors, C., Lostutter, T. W., Cronce, J. M., & Larimer, M. E. (2002). Exploring college student gambling motivation. *Journal of Gambling Studies,**18*(4), 361–370. 10.1023/A:1021065116500 12514915 10.1023/A:1021065116500 PMC1797802

[CR35] Rockloff, M., Russell, A. M. T., Greer, N., Lole, L., Hing, N., & Browne, M. (2021). Young people who purchase loot boxes are more likely to have gambling problems: An online survey of adolescents and young adults living in NSW Australia. *Journal of Behavioral Addictions,**10*(1), 35–41. 10.1556/2006.2021.00007 33625382 10.1556/2006.2021.00007 PMC8969855

[CR36] Savolainen, I., Sirola, A., Vuorinen, I., Mantere, E., & Oksanen, A. (2022). Online communities and gambling behaviors—A systematic review. *Current Addiction Reports,**9*(4), 400–409. 10.1007/s40429-022-00430-x 10.1007/s40429-022-00430-x

[CR37] Sirola, A., Kaakinen, M., & Oksanen, A. (2018). Excessive gambling and online gambling communities. *Journal of Gambling Studies,**34*(4), 1313–1325. 10.1007/s10899-018-9772-0 29623505 10.1007/s10899-018-9772-0

[CR38] Sirola, A., Savela, N., Savolainen, I., Kaakinen, M., & Oksanen, A. (2021). The role of virtual communities in gambling and gaming behaviors: A systematic review. *Journal of Gambling Studies,**37*(1), 165–187. 10.1007/s10899-020-09946-1 32306232 10.1007/s10899-020-09946-1 PMC7882555

[CR39] Sleczka, P., & Romild, U. (2021). On the stability and the progression of gambling problems: Longitudinal relations between different problems related to gambling. *Addiction,**116*(1), 116–125. 10.1111/add.15093 32307761 10.1111/add.15093

[CR40] Stevens, M., Gupta, H., & Flack, M. (2019). *2018 Northern Territory Gambling Prevalence and Wellbeing Survey*. https://industry.nt.gov.au/__data/assets/pdf_file/0010/959176/2018-nt-gambling-prevalence-wellbeing-survey.pdf

[CR41] Stevens, M., Thoss, M., & Barnes, T. (2017). *2015 Northern Territory Gambling Prevalence and Wellbeing Survey*. https://justice.nt.gov.au/__data/assets/pdf_file/0019/424135/nt-2015-gambling-prevalence-and-wellbeing-survey.pdf

[CR42] Stewart, S. H., & Zack, M. (2008). Development and psychometric evaluation of a three-dimensional Gambling Motives Questionnaire. *Addiction,**103*(7), 1110–1117. 10.1111/j.1360-0443.2008.02235.x 18554344 10.1111/j.1360-0443.2008.02235.x

[CR43] Tabachnick, B. G., & Fidell, L. S. (2012). *Using multivariate statistics* (6th ed.). Pearson.

[CR44] Thomas, S. L., Bestman, A., Pitt, H., Deans, E., & Randle, M. J. (2015). *The marketing of wagering on social media: An analysis of promotional content on YouTube, Twitter and Facebook*. V. R. G. Foundation.

[CR45] Tomei, A., Petrovic, G., & Simon, O. (2022). Offline and online gambling in a swiss emerging-adult male population. *Journal of Gambling Studies*. 10.1007/s10899-022-10106-w 35133536 10.1007/s10899-022-10106-w PMC9653321

[CR46] Tseng, C. H., Flack, M., Caudwell, K. M., & Stevens, M. (2023). Separating problem gambling behaviors and negative consequences: Examining the factor structure of the PGSI. *Addictive Behaviors,**136*, 107496. 10.1016/j.addbeh.2022.107496 36174423 10.1016/j.addbeh.2022.107496

[CR47] Wood, R. T., & Williams, R. J. (2011). A comparative profile of the Internet gambler: Demographic characteristics, game-play patterns, and problem gambling status. *New Media & Society,**13*(7), 1123–1141. 10.1177/1461444810397650 10.1177/1461444810397650

[CR48] Yi, S., Stewart, M., Collins, P., & Stewart, S. H. (2015). The activation of reward versus relief gambling outcome expectancies in regular gamblers: Relations to gambling motives. *Journal of Gambling Studies,**31*, 1515–1530. 10.1007/s10899-014-9474-1 24916965 10.1007/s10899-014-9474-1

